# A Novel Monitoring System (AUT FIT) for Anthropometrics and Physical Fitness in Primary School Children in Austria: A Cross-Sectional Pilot Study

**DOI:** 10.3390/sports10010004

**Published:** 2021-12-24

**Authors:** Gerald Jarnig, Johannes Jaunig, Reinhold Kerbl, Rodrigo Antunes Lima, Mireille N. M. van Poppel

**Affiliations:** 1 Institute of Human Movement Science, Sport and Health, University of Graz, 8010 Graz, Austria; johannes.jaunig@uni-graz.at (J.J.); mireille.van-poppel@uni-graz.at (M.N.M.v.P.); 2 Department of Pediatrics and Adolescent Medicine, LKH Hochsteiermark, 8700 Leoben, Austria; reinhold.kerbl@kages.at; 3 Research, Innovation and Teaching Unit, Parc Sanitari Sant Joan de Déu, CIBERSAM, Sant Boi de Llobregat, 08830 Barcelona, Spain; rodrigoantlima@gmail.com

**Keywords:** test battery, monitoring, children, school, body mass index, waist-to-height ratio, weight classification, physical fitness, health-related fitness, motor fitness

## Abstract

Monitoring of anthropometric and physical fitness parameters in primary school children is important for the prevention of future health problems. Many of the existing test batteries that are useful for monitoring require expensive test materials, specialized test administrators, and a lot of space. This limits the usefulness of such tests for widespread use. The aim of this pilot study was to design and evaluate monitoring tools for anthropometrics and physical fitness tests in primary schools, called AUT FIT. The test battery consists of height, weight, and waist circumference measurement and eight fitness tests (6 min run, V sit-and-reach, jumping sideways, standing long jump, medicine ball throw, 4 × 10 m shuttle run, ruler drop, single leg stand). Data of 821 children aged 7 to 10 years were gathered. Most AUT FIT tests showed excellent test–retest and interrater reliability and were easy to implement. Criterion-related validity was evident by a strong correlation between physical education teacher rankings and rank scores for motor fitness. Nationwide implementation in the Austrian school system could be an important component for monitoring and improving the health and fitness of primary school children.

## 1. Introduction

The lifelong health benefits of adequate physical activity in childhood are well established [1,2,3,4,5,6,7]. Interactions exist between physical activity, physical fitness, motor competence, the human psyche, and body weight, and their interplay has important effects on health [8,9,10,11,12,13,14].

In older studies, the level of physical fitness was defined as the sum of the performance of cardiorespiratory endurance, muscular endurance, muscular strength, speed, flexibility, full body coordination, balance, and body composition and was divided into health-related and motor fitness [15,16].

Health-related fitness (HRF) includes cardiorespiratory endurance, muscular strength, flexibility, and body composition [15,16]. Recent research views body composition as the result of relationships among HRF, physical activity, motor competence, and perceived motor competence, and thus excludes body composition as a component of HRF [11]. In addition, studies reported a weak relationship between health and flexibility [17,18] and showed that in youth, HRF is well represented by a three-component model that includes cardiorespiratory endurance, muscular endurance, and muscular strength [19].

Motor fitness includes all components described in the context of physical fitness except body composition [15,16,20]. However, balance is not a component of motor fitness, but is also considered a strong predictor of children’s spatial and proportional reasoning skills, with implications for many other milestones in childhood development [21].

There are several single motor tests available that can be used to assess components of physical fitness [22]. Likewise, there are some test batteries that allow us to assess health-related or motor fitness on the construct level. FitnessGram from the USA [23,24] and the Indares project from the International Database for Research and Educational Support [25] are examples of test batteries that allow us to assess parameters of HRF. The ALPHA-FIT test battery (an adapted version of the Eurofit test battery introduced in 1983) [26], the German motor test [27], the Czech UNIFITTEST (6–60) test battery [28], the Düsseldorf model [29], and the Identification and Prevention of Dietary and Lifestyle-Induced Health Effects in Children and Infants (IDEFICS) study [30,31] are some of the test batteries established in Europe that allow assessment of motor fitness.

Most of these test batteries are often associated with relatively high cost and require special test materials and a lot of space as well as intensive instruction of the test personnel [32]. This makes it difficult to implement them in day-to-day school life.

Newly designed monitoring tools should achieve two main goals. First, they should be able to detect deficiencies or problematic development in body composition and HRF at an early stage in order to counteract them with targeted measures. Second, the assessment of motor fitness should allow scouting of talented children for specialized sports schools and sports clubs. The aim of this pilot study was to design monitoring tools for anthropometrics and physical fitness that can be organized without high additional cost, require limited space, and can be carried out with simple instructions for the test personnel.

## 2. Materials and Methods

### 2.1. Design

This is a cross-sectional study based on the baseline measurements from a randomized controlled trial to evaluate the effects of an intervention in primary schools on the fitness and health status of children. The baseline measurements were conducted in September 2019. In early 2020, the intervention program had to be discontinued due to the COVID-19 pandemic. As part of this study, an Austrian monitoring system for anthropometrics and physical fitness was developed, the Austrian fitness monitoring tools for schoolkids (AUT FIT). The study was approved by the Research Ethics Committee of the University of Graz, Styria, Austria (GZ. 39/23/63 ex 2018/1/9) and has been registered in the German Clinical Trials Registry (ID DRKS00023824).

### 2.2. Selection of Schools and Participants

Using a random number generator, 12 of 39 primary schools in the urban and rural districts of Klagenfurt, Austria, were selected. All schools agreed to participate in the study. The following inclusion criteria were defined: children had to be between 7 and 10 years old at the beginning of the study and had to be able to perform all physical activity motor tests without limitations. In spring 2019, we invited all 1013 children attending the 12 schools to participate. Before the baseline measurements, 860 (85%) legal guardians gave written consent for their children to participate.

### 2.3. Procedures

Measurement of anthropometrics and fitness status was performed by trained members of the research team and took place in the schools during physical education (PE) lessons (the time sequence is explained in Appendix A.1 and Table A1). Information on the age and sex of the children was collected by the school teachers.

### 2.4. AUT FIT Monitoring Tools

AUT FIT was constructed to assess, in the simplest way possible, the anthropometrics and physical fitness status of school children based on three monitoring tools (Table 1).

Monitoring tools 1 (Mt1) and 2 (Mt2) assess body shape. Height (cm) was measured to the nearest 0.1 cm using a SECA 213 stadiometer. Weight (kg) was measured to the nearest 0.1 kg using a Bosch PPW4202/01 body scale, and waist circumference (cm) was measured to the nearest 0.1 cm using a GIMA 27,343 body tape measure.

#### 2.4.1. Weight Status (Mt1)

Standardized body mass index (BMI) was used for classification into weight classes (see Section 2.5). For calculation of crude BMI, body weight in kilograms was divided by height squared in meters.

#### 2.4.2. Estimate of Visceral Adiposity (Mt2)

Waist circumference was measured at the end of the breathing-out phase with a body tape measure at the level of the navel. The measurement was performed twice and the mean value from both measurements was recorded in the overall assessment. For the assessment of visceral adiposity, the waist-to-height ratio (WHtR) was used, calculated by dividing waist circumference in centimeters by height in centimeters.

#### 2.4.3. Physical Fitness (Mt3)

Monitoring tool 3 (Mt3) assesses physical fitness, which is categorized into health-related fitness (Mt3-A) and motor fitness (Mt3-B). Cardiorespiratory endurance, muscular endurance, and muscle strength were measured to assess health-related fitness [19]. Cardiorespiratory endurance, muscular endurance, muscle strength, flexibility, speed, and balance data were used to assess motor fitness [15,16].

- Cardiorespiratory endurance

The 6-min run (6 MR) was used to analyze cardiorespiratory endurance [22,29]. The children were instructed to run as far as possible within 6 min. The test was performed on the playgrounds and sports fields of the schools. A square (6 × 18 m) was marked out with sports poles, then the four corner poles were moved 0.5 m inward. The children had to run around the marked square. A group of 6 to 7 children performed the test simultaneously and their running distance was measured in meters.

- Muscular endurance and full body coordination

Jumping sideway (JS) was tested to assess muscular endurance [33] and full body coordination [22]. The test instructor marked an area on the ground (100 × 50 cm), which was divided into two squares (50 × 50 cm) with marking tape. The children stood with both legs in the middle of one square and jumped for 15 s with both legs between the squares after the start command. The aim was to complete as many jumps as possible without touching the marker. If the child touched the marker, this jump was not counted. The test instructor counted the number of valid jumps, and each jump over the center line was counted as one jump attempt. Each child had two scoring attempts, and the average of the number of valid jumps from both attempts was used.

- Lower body strength

The standing long jump (SLJ) provides an assessment of lower body muscle strength [22] and is considered as an index for the general assessment of muscular fitness in children [34]. The children had to jump as far as possible with both legs from a starting line, and the shortest distance between the start line and the child’s heel contact with the ground was measured to the nearest cm using a tape measure. Three scoring attempts were performed, and the longest of the three jumps was used.

- Upper body strength

The 1 kg medicine ball throw (MB1kg) was used to measure upper body muscle strength [22]. Each child stood on a starting line holding a 1 kg medicine ball with both hands, the ball touching their chest, then threw the ball with both hands as far forward as possible. The shortest distance between the starting line and the ball’s contact with the ground was measured to the nearest cm using a tape measure. Each child had two attempts to throw the ball, and the longest throw was considered.

- Flexibility

Flexibility was measured using the sit-and-reach test. To perform the classical sit-and-reach test, an expensive test box is needed. Therefore, the V sit-and-reach test (VSR) was chosen, which can be performed using a tape measure and marking tape. The tape measure was fixed to the ground and a heel line was marked with tape. The children sat down on ground, with feet spread 30 cm apart and heels placed at the heel line, then placed one hand on top of the other and slowly reached forward as far as they could. The distance between the heel line and the maximum position reached with the fingertips that could be held for two seconds was noted. Each child had two scoring attempts, and the longest reach was used in the overall assessment. To use reference values of the classical sit-and-reach test, 15 cm was added to the scoring attempt [35,36,37].

- Action speed

To assess the children’s action speed, a shuttle running test (4 × 10 SHR) was performed [2]. Two lines (start line and turning line) at a distance of 10 m were marked on the ground. Two objects (O1 and O3) were placed behind the turning line and one easily graspable object (O2) was placed in front of the start line. The children had to run from the start line across the turning line, pick up O1, run back across the start line, and put down O1. They then picked up O2, completed the run, ran across the turning line, put down O2, picked up O3, and ran across the start line with it. The children were instructed to complete this test as quickly as possible. Two scoring attempts were made and the time was measured to the nearest 0.01 s using a stopwatch. Each child had two attempts, and the fastest run was considered.

- Reaction speed

In order to test reaction speed, a ruler drop test (RD) was performed [33]; for this, a ruler drop stick was constructed (Methods S1) and held by the test instructor. Each child formed an angle of 45° between the thumb and the outstretched fingers, and the test instructor held the ruler drop stick centrally in this area. The zero point was held at the level of the bottom of the thumb, and the test instructor dropped the stick within three seconds after the command “Ready”. The distance in cm that the stick fell was recorded. Each child had one test attempt and five scoring attempts. The best and worst attempts were eliminated from the evaluation, and the average value was calculated from the remaining three attempts and recorded in the overall evaluation. For simplicity, a straight ruler can be used instead of the ruler drop stick construction described in Methods S1.

- Balance

Existing balance tests are very time intensive; therefore, the standard single-leg stand (SLS) test [22], where each leg is assessed for 1 min, was adopted. The children were instructed to stand with one leg on a thin wooden plank, keeping their hands on their hips, and hold this position for as long as possible, for a maximum of 45 s. The test was performed twice with each leg (left, SLS-L, and right, SLS-R), and the best score (in seconds) for each leg was considered. If the child reached the maximum value (45 s) on the first attempt, a second attempt was not performed with the same leg. Exact details about the construction of the test device are described in the Supplementary Materials (Methods S2).

#### 2.4.4. Procedure

Except the 6MR, all tests were performed in the gym or physical education room of the primary schools. The children completed all tests barefoot in sportswear, except the 6MR, which was done in sneakers. Each test was explained verbally to the children before beginning and visually demonstrated by the test instructor.

The tests were completed over four PE lessons in September 2019 and were carried out by trained test instructors. Anthropometric values and balance were measured in the first PE lesson. Flexibility, action speed, and full body coordination were measured in the second PE lesson. Reaction speed, muscular endurance, and muscle strength were measured in the third PE lesson. Cardiorespiratory endurance was assessed in the fourth PE lesson. Children who were absent at the time of testing had the opportunity to make up the missing tests during additional PE lessons.

To perform AUT FIT, a body scale (Mt1-weight), a body tape measure (Mt2), marking cones (6MR), a tape measure (6MR, SLJ, MB1kg, 4 × 10 SHR), a meter stick (Mt1-height, VSR, RD) marking tape (VSR, JS, SLJ, MB1kg, 4 × 10 SHR), three easy-to-grab items (4 × 10 SHR), a measuring construction made of wood for the balance test (Methods S2), and a stopwatch are needed. All of these items are in the typical inventory of an elementary school or can be obtained easily and inexpensively.

### 2.5. Standardization and Classification

#### 2.5.1. Weight Classification

For the standardization of BMI and classification of weight, national reference values were used [38]. National reference data were expressed in BMI centile curves (i.e., equicurves, in this report named EQUI BMI) [38]. The absolute BMI values were converted to EQUI BMI values using the procedure described in Mayer et al. [38] (based on Cole et al. [39]). EQUI BMI curves were used to project actual BMI to cut-off values at age 18 years in order to classify the children’s weight in five categories (underweight < 18.5 kg/m^2^, normal weight = 18.5 to 25.0 kg/m^2^, overweight ≥ 25.0 kg/m^2^, obese ≥ 30.0 kg/m^2^, morbidly obese ≥ 35.0 kg/m^2^) (Table S1).

#### 2.5.2. Waist-to-Height Ratio

The WHtR is a quick and an easy screening tool that can provide a simple overview of obesity and the associated cardiovascular risk. A WHtR value of 0.5 can be used for a simple general assessment of potentially increased health risk due to abdominal obesity [40]. Ashwell and Gibson proposed cut-off values of 0.5 and 0.6 to classify WHtR into no (≤0.5), increased (0.5 to 0.6), and high (≥0.6) health risk (Table S1) [41].

#### 2.5.3. Fitness Tests

Health-related fitness (Mt3-A) and MF (Mt3-B) were assessed using a nine-point rating, and the calculation process is based on seven steps, which are described in detail in the appendix (Appendix A.2, Table A2).

#### 2.5.4. Test–Retest Reliability

To assess the test–retest reliability of the four monitoring tools, 17 children in one class were tested twice by the same test instructor, with a one-week period between the test days.

#### 2.5.5. Interrater Reliability 

To assess the interrater reliability of the four monitoring tools, 18 children in one class were tested and assessed independently by two test instructors at the same time.

#### 2.5.6. Criterion Validity

To estimate the usefulness of the Mt3-B monitoring tool and to provide criterion validity, the physical education teacher in one of the tested classes was asked to complete an assessment according to his perception. He ranked the children’s motor fitness (for boys and girls separately), and the number of boys (n = 10) and girls (n = 10) who were assessed determined the highest score to be achieved, with the most points given for the best performance and one point for the worst performance. The teacher was trained in physical education, had extensive experience in grading the physical performance of students, and was not informed about the AUT FIT results from Mt3-B.

For this class, a parallel ranking was created (using the same method as for the physical education teacher assessment) based on the results from Mt3-B.

### 2.6. Statistical Analysis

Continuous variables are reported as mean (M) and standard deviation (SD), and categorical variables as absolute value (n) and percentage (%) for descriptive statistics. No imputation of the data was performed. All statistical analyses were performed in SPSS 27.0 (IBM SPSS Statistics 27, IBM, New York, USA) with a significance level of *p* < 0.05.

For both test–retest and interrater reliability, a two-way mixed intraclass correlation coefficient (ICC) based on single measures and absolute agreement was calculated for the raw scores of each physical fitness test. The strength of ICC was classified according to Koo and Li [42] as poor (ICC < 0.50), moderate (0.50 ≤ ICC ≤ 0.75), good (0.75 < ICC ≤ 0.90), and excellent (ICC > 0.90) reliability.

Spearman correlation coefficient (rho) was calculated between the rankings based on Mt3-B and the physical education teacher, as well as between the monitoring tools and the individual fitness tests. The strength of the correlations was classified according to Cohen [43], with a weak correlation classified as rho ≥0.1, a medium strong correlation as rho ≥0.3, and a strong correlation as rho ≥0.5.

## 3. Results

### 3.1. Results of AUT FIT

In September 2019, 824 children participated in anthropometric and fitness measurements. Three children did not participate in all measurements and were excluded from the analysis; thus, data from 821 children were used for analysis. The mean age of the study population was 8.3 years (0.7 SD), and 419 (51.0%) were girls (Table 2). The results from Mt1 show that 124 children (15.1%) were overweight or obese. In Mt2, 119 children (14.5%) showed increased health risk and 21 (2.6%) high health risk based on WHtR (Table 2).

The mean values of SDS and z-score calculations of JS are very high when using the German reference values (M = 2.16) [27]. Similar results are observed when using the Indian (M = 2.02) [44] or German (M = 1.18) [45] reference values for RD (Table S2).

VSR and RD showed no correlation, and the other fitness measurements showed weak or moderate correlation with both anthropometric monitoring tools (Mt1 and Mt2) (Table S3).

Comparing the results of the individual fitness tests between children in different weight categories, for MB1kg, there was no significant difference between children with normal weight and those with overweight or obesity (normal weight vs. overweight: *p* = 0.11; normal weight vs. obesity: *p* = 0.76; normal weight vs. extreme obesity: *p* > 0.99; Table S4, Figure S1). All other test results were poorer for children with overweight or obesity compared to those with normal weight (Table S4, Figure S1). Similar results were found when comparing Mt2 and the different motor fitness tests; children with lower health risk had better results on the fitness tests, except for MB1kg, on which they had poorer results compared to children with high health risk (Table S5, Figure S2).

### 3.2. Reliability of AUT FIT

#### 3.2.1. Test–Retest Reliability

The raw scores showed good or excellent test–retest reliability for eight items (weight, height, waist circumference, 6MR, SLJ, VSR, 4 × 10 SHR, and SLS-L). Moderate test–retest reliability was observed for MB1kg and SLS-R, and poor test–retest reliability was observed for JS. The test–retest reliability for the RD was poor (Table 3).

#### 3.2.2. Interrater Reliability

The results showed excellent (ICC ≥ 0.90) interrater reliability for all items except VSR (ICC = 0.88) and RD (ICC = 0.18) (Table 4).

### 3.3. Validity of AUT FIT

**Figure 1:**
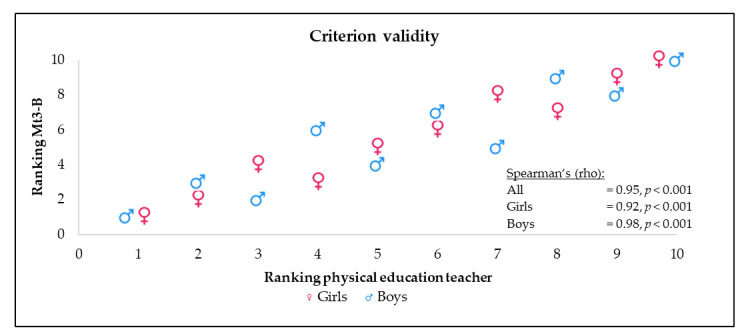
Criterion validity of motor fitness monitoring tool. Correspondence between rankings of 10 girls and 10 boys from one primary school class on a scale from worst (1) to best (10) motor fitness by assessment of PE teacher and monitoring tool 3B (Mt3-B).

Criterion validity was evident by a high Spearman’s correlation coefficient (rho = 0.95, *p* < 0.001) between the two rank scores (teacher ranking and ranking from Mt3-B), which was found to be somewhat lower for girls (rho = 0.92, *p* < 0.001) than boys (rho = 0.98, *p* < 0.001) (Figure 1).

## 4. Discussion

The monitoring tools of AUT FIT offer the possibility to conduct a multifaceted assessment of child development in terms of anthropometrics and fitness. With the data collected by AUT FIT, it may be possible to identify undesirable development at an early stage. Since the tests that make up this battery may be valid for older age groups, it might be possible to initiate effective countermeasures and monitor their effects in the long term. This approach already exists, at least in early childhood, in Austria and many other countries. Parameters of human development are routinely observed, starting before birth, and regulated by law up to the age of 60 months through mandatory examinations described in the “mother–child passport” [46,47,48,49]. After children enter the school system, monitoring is carried out by school physicians as part of the annual school examinations [50,51,52]. Although monitoring of cognitive competency is obligatory in the Austrian school system [53], a systematic examination of health-related fitness or motor fitness is currently not integrated into the system [54].

Some countries have routinely collected children’s anthropometric and fitness data nationwide for years [23,55,56,57,58], or implemented nationwide monitoring systems in primary schools, such as the German Health Interview and Examination Survey for Children and Adolescents (KiGGS) [59] and the Slovenian National Surveillance System for physical and motor development (SLOFit) [60]. SLOFit is a positive example of how a monitoring system in schools can reduce the prevalence of obesity and at the same time increase the physical fitness level of children through interventions based on its results [61].

Based on our findings in this pilot study, some of the fitness tests need to be replaced. In general, the RD test showed very poor reliability and a low correlation with physical fitness. In addition, only old reference values were available for this test, and it is not clear whether these data continue to reflect current trends [44,45]. These results render the test inadequate for further use.

The JS test resulted in very high z-scores, with a mean value of more than 2. This indicates that the children in our sample performed on average two standard deviations better than the reference sample. Various manuals of the JS test can be found in the literature, and we used the protocol described by Bös [27]. In another study using this test procedure, similarly high mean z-scores were found, as in our sample [62]. Therefore, it is suggested to use a slightly different protocol for the JS test, which uses a wooden stick [63] or a jump rope [64] to define the midline.

The new monitoring tools (Mt3-A and Mt3B) showed good results on the main quality criteria. Reliability was very satisfactory based on excellent interrater and test–retest reliability. The present study is a pilot study for the development of such a tool, and components of Mt3-A and Mt3-B are still being adapted until the final development stage of AUT FIT is completed. Criterion-related validity was demonstrated by the strong correlation between physical education teacher rankings and Mt3-B scores.

AUT FIT has several strengths and limitations. One strength is that almost all of the data collected in AUT FIT come from established and widely used motor tests that are easy to perform without requiring much additional cost, space, or time. Seven of the eight items are existing standardized tests that have been used for decades in a plethora of test batteries (FitnessGram [24], CNSPFS [56], AFEA [55], GTO [58], PFAAT [57], SLOFit [60], ALPHA [26], GMT [27], DÜMO [29]) and extensively tested for their validity. Over four physical education lessons, it was possible to collect a broad panel of anthropometric and fitness-related parameters. A major strength of AUT FIT is that no material or aspect of the test battery is culturally specific to Austrian children. AUT FIT has the potential to be used in the economically weakest regions of the world, since a good proportion of the test material could be made available in schools even in developing countries, and a vast majority of schools worldwide offer physical education lessons for this age group.

A limitation is that no national norms are available for the fitness tests so far, and some of the available international norms may be outdated. Another limitation is that AUT FIT is still in the development phase and the adaptations described above remain to be done, and for this reason confirmatory factor analysis of the proposed models is still lacking. Specific test limitations are described in the Methods and Discussion sections.

## 5. Conclusions

Our study shows that the AUT FIT monitoring tool can be easily implemented in primary schools in Austria. The tool can be easily further improved by adapting some fitness tests. The authors urgently recommend implementation of the adapted monitoring tools in schools in Austria and evaluation of the impact of the system on the anthropometrics and fitness of primary school children in the long term. This is also in view of the striking changes seen as a consequence of the COVID-19 pandemic and associated measures such as school closures and sport restrictions [65].

## Figures and Tables

**Table 1:** Detailed overview of monitoring tools in AUT FIT.

AUT FIT				Monitoring			
				Tool 1 (Mt1)	Tool 2 (Mt2)	Tool 3 (Mt3)	
						Mt3-A	Mt3-B
Anthropometrics	Weight (kg)		BMI (kg/m^2^)	Weight classification by BMI			
	Height (cm)						
			WHtR		Estimate of visceral adiposity		
	Waist circumference (cm)						
Physical fitness	Cardiorespiratory fitness		6 MR (m)			Health-related fitness	Motor fitness
	Muscular endurance/full-body coordination		JS (N)				
	Muscular strength	Lower body strength	SLJ (cm)				
		Upper body strength	MB1kg (cm)				
	Flexibility		VSR (cm)				
	Speed	Action speed	4 × 10 SHR (s)				
		Reaction speed	RD (cm)				
	Balance		SLS-L (s)				
			SLS-R (s)				

Mt3-A, monitoring tool for health-related fitness; Mt3-B, monitoring tool for motor fitness; BMI, body mass index, WHtR, waist-to-height ratio; 6 MR, 6 min run; JS, jumping sideways; SLJ, standing long jump; MB1kg, medicine ball throw (1 kg); VSR, V sit-and-reach test; 4 × 10 SHR, 4 × 10 m shuttle run; RD, ruler drop test; SLS-L, single leg stand test, left; SLS-R, single leg stand test, right. The four monitoring tools are indicated with different colors.

**Table 2:** Overall results for AUT FIT monitoring tools.

Variable	Classification	All (n = 821)
Age (years)		8.3 (0.7)
Weight (kg)		29.8 (7.2)
Height (cm)		132.1 (6.7)
Waist circumference (cm)		60.9 (8.1)
BMI (kg/m^2^)		16.9 (2.9)
EQUI BMI		22.2 (3.5)
WHtR		0.46 (0.05)
6MR (m)		913 (140)
JS (N)		31.7 (7.1)
SLJ (cm)		124 (20)
MB1kg (kg)		343 (73)
VSR (cm)		17.4 (8.5)
4 × 10 SHR (s)		15.0 (1.5)
RD (cm)		17.6 (8.1)
SLS-L (s)		22.7 (16.4)
SLS-R (s)		26.2 (15.9)
Mt1: Weight classification (N (%))	Underweight	52 (6.4%)
	Normal weight	645 (78.6%)
	Overweight	89 (10.8%)
	Obese	27 (3.3%)
	Morbidly obese	8 (1.0%)
Mt2: Health risk (N (%))	No health risk	681 (82.9%)
	Increased health risk	119 (14.5%)
	High health risk	21 (2.6%)
Mt3-A: Health-related fitness level (N (%))	Low performance	92 (11.2%)
	Average performance	516 (62.9%)
	Good performance	213 (25.9%)
Mt3-B: Motor fitness level (N (%))	Low performance	48 (5.8%)
	Average performance	639 (77.8%)
	Good performance	134 (16.3%)

Data are mean (SD) or N (%). AUT FIT, Austrian fitness monitoring tools for primary schoolkids; BMI, body mass index; EQUI BMI, equivalent BMI based on Austrian reference centile curves passing through adult BMI values (Mayer et al., 2015); WHtR, waist-to-height ratio; 6MR, 6 min run; JS, jumping sideways; SLJ, standing long jump; MB1kg, medicine ball throw (1 kg); VSR, V sit-and-reach test; 4 × 10 SHR, 4 × 10 m shuttle run; RD, ruler drop test; SLS-L, single leg stand test, left; SLS-R, single leg stand test, right; Mt1, monitoring tool for weight classification; Mt2, monitoring tool for estimating visceral adipose tissue; Mt3-A, monitoring tool for health-related fitness; Mt3-B, monitoring tool for motor fitness. Low performance includes Mt3 classification groups poor, very weak, and weak; average performance includes below average, average, and above average; good performance includes good, excellent, and outstanding.

**Table 3:** Test–retest reliability.

Antropometrics and Fitness Tests	Test Time 1	Test Time 2	ICC* (2.1)	95% CI
Weight (kg)	39.2 (10.1)	39.1 (10.3)	0.99	0.99 to >0.99
Height (cm)	142.2 (6.4)	142.1 (6.4)	0.99	0.99 to 0.99
Waist circumference (cm)	65.3 (9.4)	64.9 (9.6)	0.97	0.92 to 0.99
6MR (m)	1004 (77)	986 (72)	0.86	0.64 to 0.95
JS (N)	30.6 (5.5)	36.1 (5.3)	0.41	−0.10 to 0.76
SLJ (cm)	142.8 (15.8)	141.5 (16.2)	0.79	0.51 to 0.92
MB1kg (s)	411 (84)	436 (76)	0.70	0.35 to 0.88
VSR (cm)	11.2 (7.5)	14.5 (6.8)	0.85	0.20 to 0.96
4 × 10 SHR (s)	13.33 (0.96)	13.38 (0.69)	0.80	0.53 to 0.92
RD (cm)	24.1 (6.1)	19.4 (5.2)	−0.07	−0.40 to 0.35
SLS-L (s)	29.0 (15.9)	29.7 (14.5)	0.81	0.55 to 0.93
SLS-R (s)	31.2 (13.8)	31.7 (15.6)	0.57	0.12 to 0.82

Data are mean (SD). *ICC model is based on single measures and absolute agreement. SD, standard deviation; ICC, intraclass correlation; CI, confidence interval; 6MR, 6 min run; JS, jumping sideways; SLJ, standing long jump; MB1kg, medicine ball throw (1 kg); VSR, V sit-and-reach test; 4 × 10 SHR, 4 × 10 m shuttle run; RD, ruler drop test; SLS, single leg stand.

**Table 4:** Interrater reliability.

Antropometrics and Fitness Tests	Rater 1	Rater 2	ICC* (2.1)	95% CI
Weight (kg)	38.5 (10.2)	38.5 (10.2)	>0.99	>0.99 to >0.99
Height (cm)	141.6 (6.9)	141.5 (6.9)	0.99	0.99 to >0.99
Waist circumference (cm)	65.8 (9.2)	65.1 (9.2)	0.96	0.90 to 0.98
6MR (m)	983 (118)	982 (118)	>0.99	>0.99 to >0.99
JS (N)	30.5 (5.3)	30.7 (5.2)	0.98	0.95 to 0.99
SLJ (cm)	140.9 (18.0)	140.6 (17.8)	0.99	0.99 to 0.99
MB1kg (s)	412 (86)	408 (83)	0.99	0.99 to 0.99
VSR (cm)	11.2 (7.5)	10.6 (7.8)	0.88	0.70 to 0.95
4 × 10 SHR (s)	13.22 (0.90)	13.37 (0.95)	0.97	0.84 to 0.99
RD (cm)	23.6 (6.3)	18.0 (5.0)	0.18	−0.15 to 0.53
SLS-L (s)	27.6 (16.6)	27.8 (16.5)	0.99	0.99 to 0.99
SLS-R (s)	29.8 (14.7)	29.7 (14.7)	>0.99	0.99 to >0.99

Data are number or mean (SD). *ICC model is based on single measures and absolute agreement. SD, standard deviation; ICC, intraclass correlation; CI, confidence interval; 6MR, 6 min run; JS, jumping sideways; SLJ, standing long jump; MB1kg, medicine ball throw (1 kg); VSR, V sit-and-reach test; 4 × 10 SHR, 4 × 10 m shuttle run; RD, ruler drop test; SLS, single leg stand.

## Data Availability

The data presented in this study are available on request from the corresponding author. The data are not publicly available due to privacy/ethical restrictions.

## Supplementary Materials

The following are available online at www.mdpi.com/article/10.3390/sports10010004/s1, Methods S1: Sport motor Test for Speed time—ruler drop, Methods S2: Sport motor Test for Balance—single leg stand, Table S1: Classification of anthropometrics for Mt1 and Mt2, Table S2: Means of standard deviation scores and z-scores of physical fitness test for total sample, Table S3: Spearman correlations between the AUT-FIT monitoring tools and nine-point rating of each physical fitness test ratings, Table S4: Kruskal-Wallis Test for differences in physical fitness between weight categories, Table S5: Kruskal-Wallis Test between waist-to-height ratio categories and physical fitness tests, Table S6: Detailed overview of reference values used for the comparison for single sport motor tests, Figure S1: Motor fitness according to weight classification. For comparing the weight classifications with the physical fitness tests, the three weight classifications of underweight are combined into one group with EQUI BMI < 18.5; 6MR = results of 6 min run recorded in step four classification of physical fitness, JS = results of jumping sideways recorded in step four classification of physical fitness, SLJ = results of standing long jump recorded in step four classification of physical fitness, MB1kg = results of medicine ball throw (1 kg) recorded in step four classification of physical fitness, VSR = results of V sit-and-reach recorded in step four classification of physical fitness, 4 × 10 SHR = results of 4 × 10 m shuttle run recorded in step four classification of physical fitness, RD = results of ruler drop recorded in step four classification of physical fitness, SLS = results of single leg stand recorded in step five classification of physical fitness. u.w. = underweight, n.w. = normal weight, ov. = overweight, o. = obesity, m.o. = morbid obesity; EF. = Effect size (according to Cohen) for pairwise Comparisons of Kruskal-Wallis Test between weight classification and physical fitness tests, u.w. = underweight, n = normal weight, ov. = overweight, o. = obesity, m.o. = morbid obesity. Figure S2: Motor fitness according to (three-level) waist-to-height classification. For comparing the weight classifications with the physical fitness tests, the three weight classifications of underweight are combined into one group with EQUI BMI < 18.5; 6MR = results of 6 min run recorded in step four classification of physical fitness, JS = results of jumping sideways recorded in step four classification of physical fitness, SLJ = results of standing long jump recorded in step four classification of physical fitness, MB1kg = results of medicine ball throw (1 kg) recorded in step four classification of physical fitness, VSR = results of V sit-and-reach recorded in step four classification of physical fitness, 4 × 10 SHR = results of 4 × 10 m shuttle run recorded in step four classification of physical fitness, RD = results of ruler drop recorded in step four classification of physical fitness, SLS = results of single leg stand recorded in step five classification of physical fitness. u.w. = underweight, n.w. = normal weight, ov. = overweight, o. = obesity, m.o. = morbid obesity; EF. = Effect size (according to Cohen) for pairwise Comparisons of Kruskal-Wallis Test between waist-to-height ratio classification and physical fitness tests, no.h.r.= no health risk, h.r.= health risk.

## Appendix A

### Appendix A.1

All data for AUT FIT were collected in one class by one person; thus, four school lessons of 50 min each were needed. When carrying out the tests, it was important that the main muscle groups used were not tested one after the other. Tests assessing endurance or speed performance (6MR, 4 × 10 SHR) were carried out at the beginning of the lesson. The endurance performance in running (6MR) was tested with 6 to 8 children at the same time, and the balance test was carried out by two children on two test devices standing parallel to each other. The remaining tests (anthropometrics, jumping sideways, standing long jump, 1 kg medicine ball throw, V-sit-and-reach test, 4 × 10 m shuttle run, ruler drop test) were assessed individually. Alternatively, it is possible to carry out the assessments by two test instructors at the same time; in this case, the data used for AUT FIT could also be collected in two school lessons.

### Appendix A.2

The assessment of health-related and motor fitness was performed in seven steps.

Step one: To ensure a valid evaluation despite the lack of Austrian reference values for the fitness tests, three standard deviation scores (SDS) or z-values were calculated for all physical fitness tests, except balance, using age- and sex-specific reference values derived from international studies. Two international reference values and the current study population were used to calculate the SDS and z-scores. To compare the results of the RD test with international reference values, the drop distance (s) in cm was doubled and divided by the gravitational constant (g = 9.81 m/s^2^). The square root of the result was taken, and in this way the achieved reaction time (RT) in milliseconds (ms) was calculated. A detailed list of all international reference values used and the calculation method (LMS method or traditional z-score standardization) is given in Table S6.

Step two: The SDS or z-scores of each fitness test were transformed into a nine-point rating. The transformation of the STA9 scores gave a distribution with a mean of 5 and a standard deviation of 2.

Step three: STA9 scores were classified by a nine-point rating: the worst athletic performance was given 1 point and the best performance 9 points [66].

Step four: For each fitness test, the mean value was calculated from the three individual nine-point rating values (own study group and two international reference values).

Step five: To include the modified balance test in the overall assessment of motor fitness, a meaningful nine-point rating was self-constructed. This involved awarding 1 point for every 5 s achieved, resulting in a maximum score of 9 points for 45 s. This assessment was performed for both legs, the scores of the two legs were added, and the results were translated into a nine-point scale (0–2 points = 1 point; 3–4 points = 2 points; 5–6 points = 3 points; 7–8 points = 4 points; 9–10 points = 5 points; 11–12 points = 6 points; 13–14 points = 7 points; 15–16 points = 8 points; 17–18 points = 9 points), which was incorporated into the overall motor fitness score.

Step six: For the overall health-related fitness score (Mt3-A), the scores recorded in cardiorespiratory fitness (6MR), muscle strength (SLJ), and muscular endurance (JS) of step four were summed. For the overall motor fitness score (Mt3-B), the scores recorded of all motor fitness tests in step four were added together.

Step seven: For the two sum scores (Mt3-A and Mt3-B) made in step six, the classification was transformed into a nine-point rating, with the worst performance given 1 point and the best performance 9 points.

**Table A1:** Carrying out AUT FIT plan.

One Test Instructor				
	**Lesson (Duration: 50 min)**			
	0–16 min	17–33 min		34–50 min
Lesson 1	W/H/WC	RD		VSR
Lesson 2	4 × 10 SHR	MB1kg		SLJ
Lesson 3	JS		BA (SLS-L + SLS-R)	
Lesson 4	6MR			
**Two Test Instructors**				
	**Lesson (Duration: 50 min)**			
	0–16 min	17–33 min		34–50 min
Lesson 1, Test instructor 1	W/H/WC	RD		VSR
Lesson 1, Test instructor 2	4 × 10 SHR	MB1kg		SLJ
Lesson 2, Test instructor 1	JS		BA	
Lesson 2, Test instructor 2	6MR			

W, weight; H, height; WC, waist circumference; RD, ruler drop; VSR, V sit-and-reach; JS, jumping sideways; 4 × 10 SHR, 4 × 10 m shuttle run; MB1kg, medicine ball throw (1 kg); SLJ, standing long jump; BA, balance; SLS-L, single leg stand, left; SLS-R, single leg stand, right; 6MR, 6 min run.

**Table A2:** Classification of fitness by nine-point rating for Mt3-A and Mt3-B.

Step 1				Step 2		Step 3	
Calculation of standard deviation scores (SDS) or traditional z-values based on own study group and two international reference values				SDS or z-scores of fitness tests are converted to nine-point scale (STA9) using inverse z-standardization		Classification of STA9 scores into a nine-point rating	
						STA9 values	Points for Mt3-A and Mt3-B
						<2.0	1
						2.0 to 3.0	2
6MR						3.0 to 4.0	3
JS						4.0 to 5.0	4
SLJ						5.0 to 6.0	5
MB1kg						6.0 to 7.0	6
VSR						7.0 to 8.0	7
4 × 10 SHR						8.0 to 9.0	8
RD						≥9.0	9
**Step 4**		**Step 5**					
Calculation of mean from three individual nine-point rating scores recorded in step three (own study group and two international reference scores)		Self-constructed nine-point rating for balance					
		Assessment of left leg		Assessment of right leg		Overall assessment of balance	
		SLS-L	Points for SLS-L	SLS-R	Points for SLS-R	Sum of SLS-L and SLS-R points and cut off points for nine-point rating of balance	Points for assessment of balance
		Balance (s)		Balance (s)			
		≤9.9	1	≤9.9	1	2	1
		10.0 to 14.9	2	10.0 to 14.9	2	2.1 to 4.0	2
		15.0 to 19.9	3	15.0 to 19.9	3	4.1 to 6.0	3
		20.0 to 24.9	4	20.0 to 24.9	4	6.1 to 8.0	4
		25.0 to 29.9	5	25.0 to 29.9	5	8.1 to 10.0	5
		30.0 to 34.9	6	30.0 to 34.9	6	10.1 to 12.0	6
		35.0 to 39.9	7	35.0 to 39.9	7	12.1 to 14.0	7
		40.0 to 45.9	8	40.0 to 45.9	8	14.1 to 16.0	8
		≥45.0	9	≥45.0	9	>16.0	9
**Step 6**		**Step 7**					
Mt3-A = Calculation of sum from mean scores of 6MR, JS, and SLJ recorded in step four	Mt3-B = Calculation of sum from mean scores of all physical fitness tests recorded in step four and points for balance in step five	Classification of sum scores (Mt3-A and Mt3-B) made in step six for overall assessment by nine-point rating					
		Cut-off points of sum for Mt3-A	Cut-off points of sum for Mt3-B	Points and description of performance for nine-point rating resulting from sum values for Mt3-A and Mt3-B			
		<5.0	<12	1		Poor	
		5.0 to 7.9	12.0 to 19.9	2		Very weak	
		8.0 to 10.9	20.0 to 27.9	3		Weak	
		11.0 to 13.9	28.0 to 35.9	4		Below average	
		14.0 to 16.9	36.0 to 43.9	5		Average	
		17.0 to 19.9	44.0 to 51.9	6		Above average	
		20.0 to 22.9	52.0 to 59.9	7		Very good	
		23.0 to 25.9	60.0 to 67.9	8		Excellent	
		≥26.0	≥68.0	9		Outstanding	

Mt3-A, assessment of health-related fitness; Mt3-B, assessment of motor; 6MR, 6 min run, JS, jumping sideways; SLJ, standing long jump; MB1kg, medicine ball throw (1 kg); VSR, V sit-and-reach; 4 × 10 SHR, 4 × 10 m shuttle run; RD, ruler drop; SLS-L, single leg stand, left; SLS-R, single leg stand, right.
