# Supplementary material for: A Novel Monitoring System (AUT FIT) for Anthropometrics and Physical Fitness in Primary School Children in Austria: A Cross-Sectional Pilot Study
Source: Sports (Basel). 2021 Dec 24;10(1):4. doi: 10.3390/sports10010004 (PMC8822895; doi:10.3390/sports10010004)
Supplement: Supplementary file 1 [file sports-10-00004-s001.zip › sports-1479398-supplementary.pdf]

sports

Supplementary material

A Novel Monitoring System (AUT FIT) for Anthropometrics and Physical Fitness in Primary School Children in Austria: A Cross-Sectional Pilot Study

Gerald Jarnig [1,*], Johannes Jaunig [1], Reinhold Kerbl [2], Rodrigo Antunes Lima [3] and Mireille N. M. van Poppel [1]

This supplementary material has been provided by the authors to give readers additional information about their work.

Methods S1. Sport motor Test for Speed time – ruler drop

Methods S2. Sport motor Test for Balance – single leg stand

Table S1. Classification of anthropometrics for Mt1 and Mt2
Table S2. Means of standard deviation scores and z-scores of physical fitness test for total sample
Table S3. Spearman correlations between the AUT-FIT monitoring tools and nine-point rating of each physical fitness test ratings
Table S4 Kruskal-Wallis Test for differences in physical fitness between weight categories
Table S5. Kruskal-Wallis Test between waist-to-height ratio categories and physical fitness tests
Table S6. Detailed overview of reference values used for the comparison for single sport motor tests
Figure S1. Motor fitness according to weight classification
Figure S2. Motor fitness according to (three-level) waist-to-height classification

Methods S1. Sport motor Test for Speed time – the ruler drop test

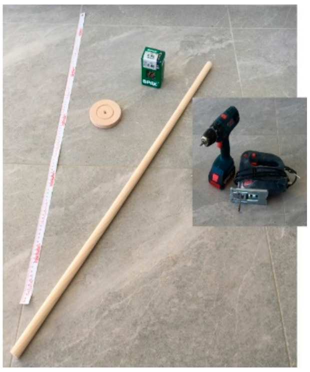

For testing, a drop rod was constructed using cheap construction materials.

Material list:
1 wooden drop rod: 100 cm long and 2.2 cm in diameter
1 wooden round plate: diameter: 10.0 cm; thickness: 12 mm
1 paper measuring tape
1 wood screw 4 cm× 45.4 cm, 5 cm long
1 transparent adhesive tape

Hand tools: Jigsaw; Hand drill

Construction instructions:
Work step 1: The wooden rod is cut to 52 cm with the jigsaw.
Work step 2: The wooden round plate is screwed onto wooden rod with wood screw and hand drill.
Work step 3: A transparent adhesive tape is used to attach the paper measuring tape to the wooden rod.

The test is carried out according to Fetz's instructions [1].

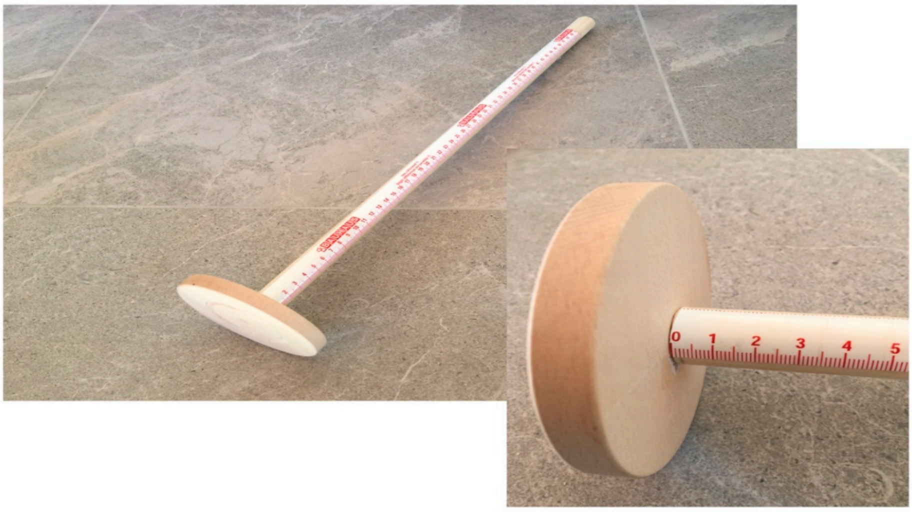

Self-constructed wooden drop stick (Jarnig Gerald, 2019)

Methods S2. Sport motor Test for Balance – single leg stand

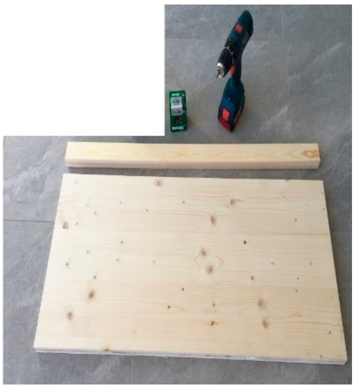

The T-rail, well-known in sports science literature [2], is adapted for use in primary schools. For barefoot testing, a T-rail with a 5 cm wide standing surface is constructed.

The materials to be used can be picked up at a local building materials market.

Material:
1 wooden board spruce 100 cm × 30 cm × 5cm
1 wooden block spruce 100 cm × 5 cm × 5 cm
3 wood screws 4 cm × 45.4 cm, 5 cm long

Hand tools: Jigsaw; Hand drill; Sanding belt for sanding the edges

Construction instructions:

Work step 1: The wooden block is screwed to the center of the wooden board using wood screws and a drill.
Work step 2: Using a hand sander, sharp cut wood edges are rounded off and made for use in schools.

Test Instruction

The test was performed with each leg twice. All testing sessions were performed without shoes. Participants were given standardized instructions. Participants were instructed to stand with arms supported at the hips, on one leg on the T-rail. The second leg was raised slightly off the ground. Due to time limitations, the maximum time to be achieved was defined as 45 s. If this time was achieved with one leg in the first test run, no further test try was performed with this leg. The test ended as soon as a participant lost the position of the supported arms at the hips, touched the ground with the second leg or reached time maximum. The best test try of each leg was used in the scoring. Between each try, the children had a minimum of 30 s's rest.

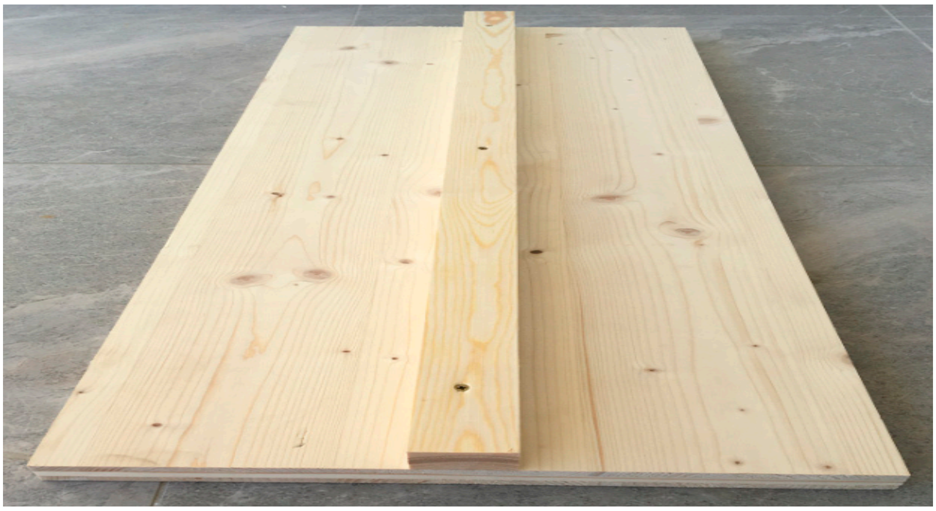

Self-constructed T-rail (Jarnig, 2019)

Table S1. Classification of anthropometrics for Mt1 and Mt2.

Mt1	**Weight Classification**	**EQUI BMI**
	underweight	< 18.50
	normal weight	18.50 to 24.99
	overweight	25.00 to 29.99
	obesity	30.00 to 34.99
	morbid obesity	≥ 35.00
Mt2	**Classification for potential health risk**	**WHtR**
	no health risk	< 0.50
	increased health risk	0.50 to 0.59
	high health risk	≥ 0.60

Mt1 = monitoring tool one, Mt2 = monitoring tool two, EQUI BMI = equivalent BMI based on Austrian reference centile curves passing through adult BMI values (Mayer et al, 2015), WHtR = waist-to-height ratio.

Table S2. Means of standard deviation scores and z-scores of physical fitness test for total sample

Fitness test	Reference values	M<br>(N = 821)
6MR	DüMo	0.46 (1.13)
	GMT	0.12 (1.08)
JS	GMT	2.16 (1.39)
	MoMo	2.14 (1.44)
SLJ	DüMo	0.15 (1.07)
	MAKFIT	0.59 (1.06)
MB1kg	DüMo	0.28 (1.00)
	KATS-K	0.04 (0.99)
VSR	MAKFIT	0.29 (1.48)
	GRE	0.34 (1.24)
4 × 10 SHR	MAKFIT	0.29 (0.83)
	MAC	−0.42 (0.94)
RD	IND	2.02 (1.67)
	MFK	1.18 (1.19)

Data are mean (SD); Data from the whole study group (821 participants); M = mean values of standard deviation scores or z-scores; SD = standard deviation, 6MR = 6 min run, JS = jumping sideways, SLJ = standing long jump, MB1kg = medicine ball throw (1 kg), VSR = V sit-and-reach, 4 × 10 SHR = 4 × 10 m shuttle run, RD = ruler drop, DüMo = Düsseldorfer Modell [3], MFK = Manual Sports Motor Tests [1], MAK = Macedonian reference values [4], GMT = German Motor Test [5], KATS-K = Karlsruher test system [6], INDIA = Indian Reference values [7], MCA = Motor Competence Assessment [8], GRE = norm values of Greek children [9], MoMo = German Motoric Modul [10].

Table S3. Spearman correlations between the AUT-FIT monitoring tools and nine-point rating of each physical fitness test ratings.

Monitoring tool / fitness test	Mt1	Mt2	Mt3-A	Mt3-B	6MR-9	JS-9	SLJ-9	MB1kg-9	VSR-9	4 × 10 SHR-9	RD-9
Mt2	0.70										
Mt3-A	−0.32	−0.28									
Mt3-B	−0.19	−0.24	0.79								
6MR-9	−0.35	−0.31	0.74	0.55							
JS-9	−0.14	−0.03	0.67	0.56	0.29						
SLJ-9	−0.23	−0.29	0.76	0.70	0.37	0.39					
MB1kg-9	−0.23	0.05	0.34	0.50	0.14	0.28	0.37				
VSR-9	−0.04	−0.08	0.20	0.45	0.08	0.17	0.25	0.13			
4 × 10 SHR-9	−0.17	−0.27	0.53	0.60	0.42	0.24	0.52	0.27	0.17		
RD-9	0.03	0.03	0.18	0.34	0.11	0.17	0.17	0.10	0.04	0.05	
SLS-9	−0.17	−0.17	0.36	0.62	0.26	0.27	0.30	0.16	0.22	0.26	0.09

Data shows Spearman correlation coefficient (ϱ) from the whole study group (821 participations); Level of significance: = P <0.05, = P <0.01, = P <0.001; Mt1 = EQUI BMI (equivalent BMI based on Austrian reference centile curves passing through adult BMI values (Mayer et al, 2015)), Mt2 = waist-to-height ratio, Mt3-A = nine point rating of monitoring tool for health-related fitness , Mt3-B = nine point rating of monitoring tool for motor fitness; 6MR-9 = nine point rating of 6 min run, JS-9 = nine point rating of jumping sideways, SLJ-9 = nine point rating of standing long jump, MB1kg-9 = nine point rating of medicine ball throw (1 kg), VSR-9 = nine

point rating of V sit-and-reach, 4 × 10 SHR-9 = nine point rating of 4 × 10 m shuttle run, RD-9 = nine point rating of ruler drop, SLS-9 = nine point rating of single leg stand.

Table S4. Kruskal-Wallis Test for differences in physical fitness between weight categories

Fitness test	TS	df	p-lvl	P^a
6MR	112.359[a]	4	***	<0.001
JS	22.955[a]	4	***	<0.001
SLJ	67.709[a]	4	***	<0.001
MB1kg	23.176[a]	4	***	<0.001
VSR	3.963[a.b]	4		0.41
4 × 10 SHR	45.884[a]	4	***	<0.001
RD	7.863[a.b]	4		0.10
SLS	25.135[a]	4	***	<0.001

In order to be able to calculate meaningful values and due to the fact that only 4 children each suffer from underweight grade 3 and underweight grade 2, the weight categories underweight grade 1, underweight grade 2 and underweight grade 3 were combined into one weight category (underweight) for this calculation. Data shows results of the total study population (n = 821), [a] = the test statistic is adjusted for ties, [b] = Multiple comparisons are not performed because the overall test does not show significant differences across samples. TS = Test Statistic, df = degree of freedom, p-lvl (P^a level) * = P <0.05, ** = P <0.01, *** = P <0.001, P^a = Asymptotic Sig.(2-sided test), 6MR = results of 6 min run recorded in step four classification of physical fitness, JS = results of jumping sideways recorded in step four classification of physical fitness, SLJ = results of standing long jump recorded in step four classification of physical fitness, MB1kg = results of medicine ball throw (1 kg) recorded in step four classification of physical fitness, VSR = results of V sit-and-reach recorded in step four classification of physical fitness, 4 × 10 SHR = results of 4 × 10 m shuttle run recorded in step four classification of physical fitness, RD = results of ruler drop recorded in step four classification of physical fitness, SLS = results of single leg stand recorded in step five classification of physical fitness.

Table S5. Kruskal-Wallis Test between waist-to-height ratio categories and physical fitness tests

Fitness test	TS	df	p-lvl	P^a
6MR	80.879[a]	2	***	<0.001
JS	18.692[a]	2	***	<0.001
SLJ	52.535[a]	2	***	<0.001
MB1kg	7.717[a]	2	*	0.021
VSR	0.703[a.b]	2		0.70
4 × 10 SHR	64.796[a]	2	***	<0.001
RD	0.407[a.b]	2		0.82
SLS	15.980[a]	2	***	<0.001

In order to be able to calculate meaningful values and due to the fact that only 4 children each suffer from underweight grade 3 and underweight grade 2, the weight categories underweight grade 1, underweight grade 2 and underweight grade 3 were combined into one weight category (underweight) for this calculation. Data shows results of the total study population (n = 821), [a] = the test statistic is adjusted for ties, [b] = Multiple comparisons are not performed because the overall test does not show significant differences across samples. TS = Test Statistic, df = degree of freedom, p-lvl (P^a level) * = P <0.05, ** = P <0.01, *** = P <0.001, P^a = Asymptotic Sig.(2-sided test), 6MR = results of 6 min run recorded in step four classification of physical fitness, JS = results of jumping sideways recorded in step four classification of physical fitness, SLJ = results of standing long jump recorded in step four classification of physical fitness, MB1kg = results of medicine ball throw (1 kg) recorded in step four classification of physical fitness, VSR = results of V sit-and-reach recorded in step four classification of physical fitness, 4 × 10 SHR = results of 4 × 10 m shuttle run recorded in step four classification

of physical fitness, RD = results of ruler drop recorded in step four classification of physical fitness, SLS = results of single leg stand recorded in step five classification of physical fitness.

Table S6. Detailed overview of reference values used for the comparison for single sport motor tests

Fitness Test	Ref. 1	Me.o.Ca.	Ref. 2	Me.o.Ca.	Ref. 3	Me.o.Ca.
6MR	OSG	A	DüMo	B	GMT	A
JS	OSG	A	GMT	A	MoMo	A
SLJ	OSG	A	DüMo	B	MAKFIT	B
MB1kg	OSG	A	DüMo	B	KATS-K	A
VSR	OSG	A	MAKFIT	B	GRE	B
4 × 10 SHR	OSG	A	MAKFIT	B	MCA	B
RD	OSG	A	MFK	A	INDIA	A

Ref.1 = Calculations in relation to own study group, Me.o.Ca. = Method of calculation, Ref. 2 = First calculation to international reference values, Ref. 3 = Second calculation to international reference values, 6MR = 6 min run, JS = jumping sideways, SLJ = standing long jump, MB1kg = medicine ball throw (1 kg), VSR = V sit-and-reach, 4 × 10 SHR = 4 × 10 m shuttle run, RD = ruler drop, OSG = Own study group, A = traditional z-score Standardization with mean = 0 and standard deviation =1, B = Calculation based on the LMS method [11], DüMo = Düsseldorfer Modell [3], GRE = norm values of Greek children [9], MFK = Manual Sports Motor Tests [1], MAKFIT = Macedonian reference values [4], GMT = German Motor Test [5], KATS-K = Karlsruher test system [6], INDIA = Indian Reference values [7], MCA = Motor Competence Assessment [8], MoMo = German Motoric Modul [10].

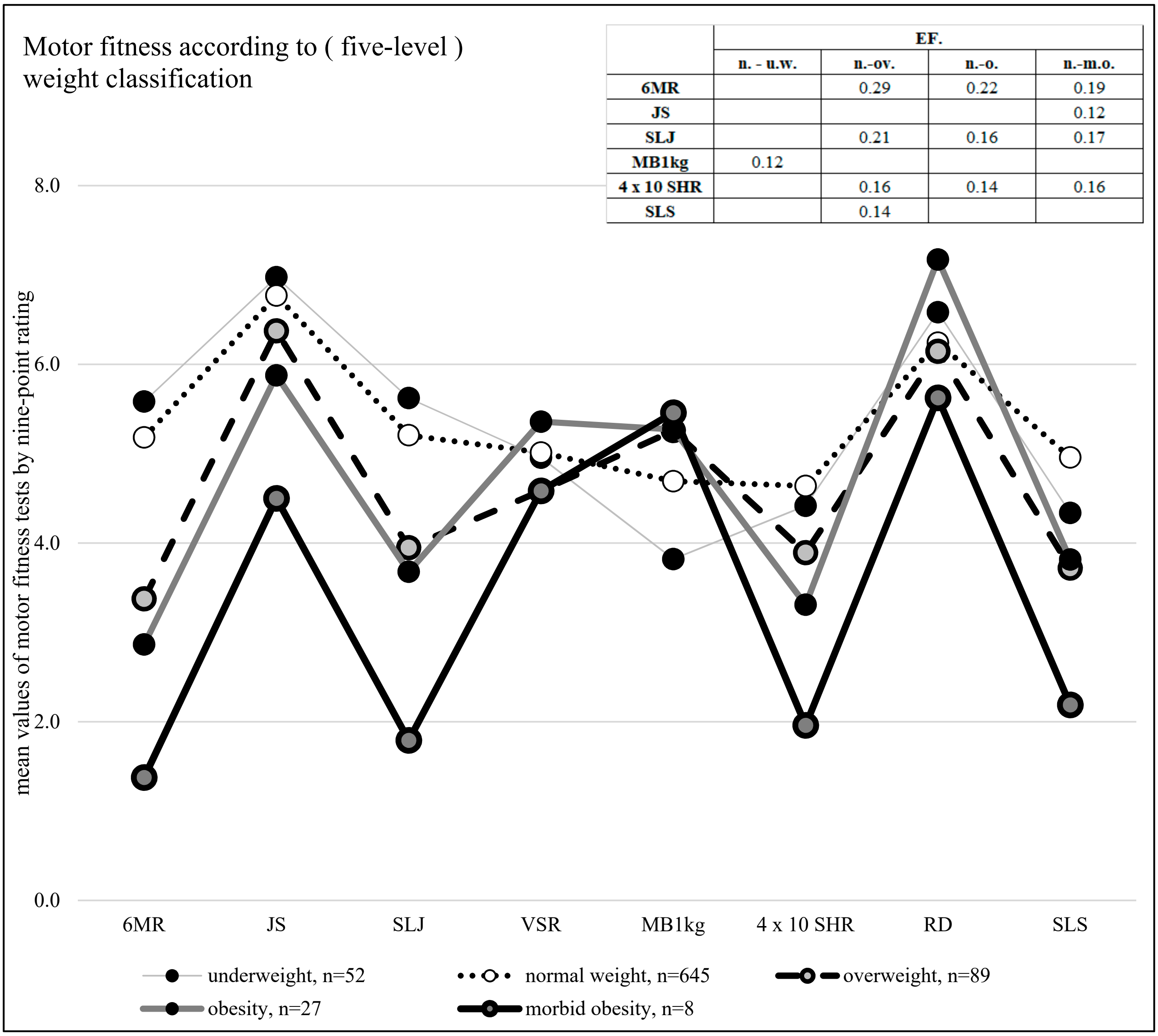

	EF.			
	n. - u.w.	n.-ov.	n.-o.	n.-m.o.
**6MR**		0.29	0.22	0.19
**JS**				0.12
**SLJ**		0.21	0.16	0.17
**MB1kg**	0.12			
**4 x 10 SHR**		0.16	0.14	0.16
**SLS**		0.14		

Figure S1. Motor fitness according to weight classification. For comparing the weight classifications with the physical fitness tests, the three weight classifications of underweight are combined into one group with EQUI BMI <18.5; 6MR = results of 6 min run recorded in step four classification of physical fitness, JS = results of jumping sideways recorded in step four classification of physical fitness, SLJ = results of standing long jump recorded in step four classification of physical fitness, MB1kg = results of medicine ball throw (1kg) recorded in step four classification of physical fitness, VSR = results of V sit-and-reach recorded in step four classification of physical fitness, 4 × 10 SHR = results of 4 × 10 m shuttle run recorded in step four classification of physical fitness, RD = results of ruler drop recorded in step four classification of physical fitness, SLS = results of single leg stand recorded in step five classification of physical fitness. u.w. = underweight, n.w. = normal weight, ov. = overweight, o. = obesity, m.o. = morbid obesity; EF. = Effect size (according to Cohen) for pairwise Comparisons of Kruskal-Wallis Test between weight classification and physical fitness tests, u.w. = underweight, n = normal weight, ov. = overweight, o. = obesity, m.o. = morbid obesity.

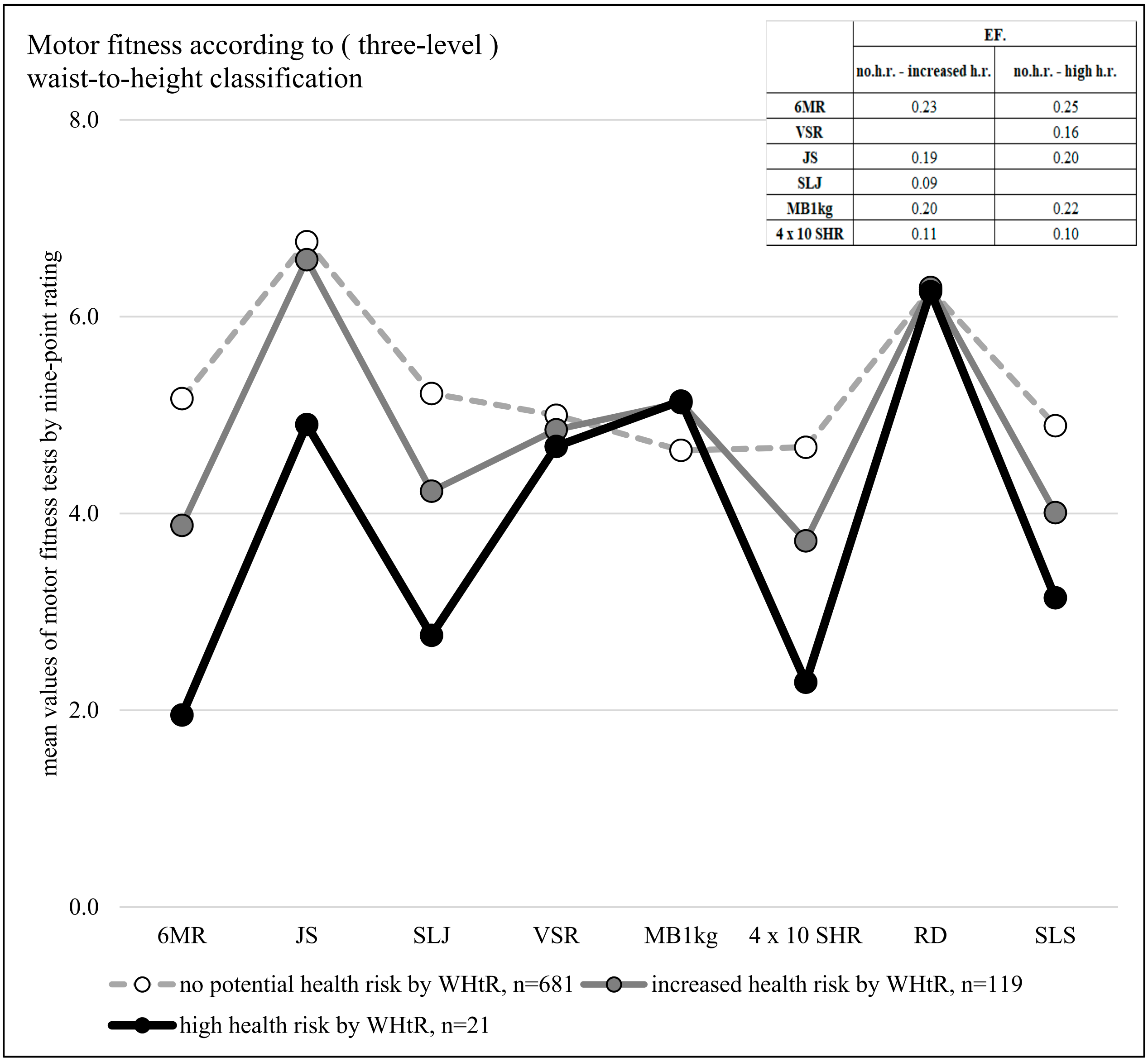

	EF.	
	no.h.r. - increased h.r.	no.h.r. - high h.r.
6MR	0.23	0.25
VSR		0.16
JS	0.19	0.20
SLJ	0.09	
MB1kg	0.20	0.22
4 x 10 SHR	0.11	0.10

Figure S2. Motor fitness according to (three-level) waist-to-height classification. For comparing the weight classifications with the physical fitness tests, the three weight classifications of underweight are combined into one group with EQUI BMI <18.5; 6MR = results of 6 min run recorded in step four classification of physical fitness, JS = results of jumping sideways recorded in step four classification of physical fitness, SLJ = results of standing long jump recorded in step four classification of physical fitness, MB1kg = results of medicine ball throw (1 kg) recorded in step four classification of physical fitness, VSR = results of V sit-and-reach recorded in step four classification of physical fitness, 4 × 10 SHR = results of 4 × 10 m shuttle run recorded in step four classification of physical fitness, RD = results of ruler drop recorded in step four classification of physical fitness, SLS = results of single leg stand recorded in step five classification of physical fitness. u.w. = underweight, n.w. = normal weight, ov. = overweight, o. = obesity, m.o. = morbid obesity; EF. = Effect size (according to Cohen) for pairwise Comparisons of Kruskal-Wallis Test between waist-to-height ratio classification and physical fitness tests, no.h.r.=no health risk, h.r.=health risk.

References

1. Fetz, Friedrich; Elmar Kornexl. *Sportmotorische Tests: Praktische Anleitung Zu Sportmotorischen Tests in Schule Und Verein*. 2, überarb. u. erw. Aufl; Bartels & Wernitz: Berlin, Germany, 1978, ISBN 387039028X.

2. Bös, K. *Handbuch Motorische Tests: Sportmotorische Tests, Motorische Funktionstests, Fragebögen zur körperlich-Sportlichen Aktivität und Sportpsychologische Diagnoseverfahren, 3., überarbeitete und erweiterte Auflage*; Hogrefe: Göttingen, Germany, 2017; ISBN 9783801723699.
3. Stemper, T.; Bachmann, C.; Diehlmann, K.; Kemper, B. *DüMo Düsseldorfer Modell der Bewegungs-, Sport- und Talentförderung: 2003 - 2018: Konzept, Normwerte, Untersuchungsergebnisse*; LIT: Berlin, Germany. 2020, ISBN 9783643147783.
4. Gontarev, S.; Kalac, R.; Velickovska, L.A.; Zivkovic, V. Physical Fitness Reference Standards in Macedonian Children And Adolescent: The Makfit Study. *Nutrición Hospitalaria* **2018**, *35*, 1275–1286, doi:10.20960/nh.1881.
5. Bös, K. *Deutscher Motorik-Test 6-18: (DMT 6-18): Manual und internetbasierte Auswertungssoftware, 2. Auflage*; Feldhaus, Edition Czwalina: Hamburg, Germany. 2016, ISBN 9783880206434.
6. Bös, K.; Opper, E.; Woll, A.; Liebisch, R.; Breithecker, D.; Kremer, B. Das Karlsruher Testsystem für Kinder (KATS-K) - Testmanual. *Haltung und Bewegung* **2001**, *21*, 4.
7. Aranha, V.P.; Moitra, M.; Saxena, S.; Narkeesh, K.; Arumugam, N.; Samuel, A.J. Motor cognitive processing speed estimation among the primary schoolchildren by deriving prediction formula: A cross-sectional study. *J. Neurosci. Rural. Pr.* **2017**, *8*, 079–083, doi:10.4103/0976-3147.193544.
8. Rodrigues, L.P.; Luz, C.; Cordovil, R.; Bezerra, P.; Silva, B.; Camões, M.; Lima, R. Normative values of the motor competence assessment (MCA) from 3 to 23 years of age. *J. Sci. Med. Sport* **2019**, *22*, 1038–1043, doi:10.1016/j.jsams.2019.05.009.
9. Tambalis, K.D.; Panagiotakos, D.B.; Psarra, G.; Daskalakis, S.; Kavouras, S.A.; Geladas, N.; Tokmakidis, S.; Sidossis, L.S. Physical fitness normative values for 6-18-year-old Greek boys and girls, using the empirical distribution and the lambda, mu, and sigma statistical method. *Eur. J. Sport Sci.* **2015**, *16*, 736–746, doi:10.1080/17461391.2015.1088577.
10. Bös, K.; Worth, A.; Opper, E.; Oberger, J.; Woll, A. *Motorik-Modul: Eine Studie zur motorischen Leistungsfähigkeit und körperlich-sportlichen Aktivität von Kindern und Jugendlichen in Deutschland: Abschlussbericht zum Forschungsprojekt, 1st ed.*; Nomos Verlag: Baden-Baden, Deutschland. 2009, ISBN 978-3-8329-4498-8.
11. Cole, T.J.; Lobstein, T. Extended international (IOTF) body mass index cut-offs for thinness, overweight and obesity. *Pediatr. Obes.* **2012**, *7*, 284–294, doi:10.1111/j.2047-6310.2012.00064.x.
